# *Mycobacterium tuberculosis* Pst/SenX3-RegX3 Regulates Membrane Vesicle Production Independently of ESX-5 Activity

**DOI:** 10.1128/mBio.00778-18

**Published:** 2018-06-12

**Authors:** Dylan W. White, Sarah R. Elliott, Evan Odean, Lynne T. Bemis, Anna D. Tischler

**Affiliations:** a Department of Microbiology and Immunology, University of Minnesota Twin Cities, Minneapolis, Minnesota, USA; b Department of Biomedical Sciences, University of Minnesota Medical School Duluth campus, Duluth, Minnesota, USA; School of Medicine, Washington University in St. Louis

**Keywords:** ESX secretion, type VII secretion, lipoproteins, tuberculosis, vesicle

## Abstract

Mycobacterium tuberculosis releases membrane vesicles (MV) that modulate host immune responses and aid in iron acquisition, although they may have additional unappreciated functions. MV production appears to be a regulated process, but *virR* remains the only characterized genetic regulator of vesiculogenesis. Here, we present data supporting a role for the M. tuberculosis Pst/SenX3-RegX3 signal transduction system in regulating MV production. Deletion of *pstA1*, which encodes a transmembrane component of the phosphate-specific transport (Pst) system, causes constitutive activation of the SenX3-RegX3 two-component system, leading to increased protein secretion via the specialized ESX-5 type VII secretion system. Using proteomic mass spectrometry, we identified several additional proteins hyper-secreted by the Δ*pstA1* mutant, including LpqH, an MV-associated lipoprotein. Nanoparticle tracking analysis revealed a 15-fold increase in MV production by the Δ*pstA1* mutant. Both hyper-secretion of LpqH and increased MV release required RegX3 but were independent of VirR, suggesting that Pst/SenX3-RegX3 controls MV release by a novel mechanism. Prior proteomic analysis identified ESX-5 substrates associated with MV. We therefore hypothesized that MV release requires ESX-5 activity. We constructed strains that conditionally express *eccD*_*5*_, which encodes the predicted ESX-5 transmembrane channel. Upon EccD_5_ depletion, we observed reduced secretion of the ESX-5 substrates EsxN and PPE41, but MV release was unaffected. Our data suggest that ESX-5 does not affect vesicle production and imply that further characterization of the Pst/SenX3-RegX3 regulon might reveal novel mechanisms of M. tuberculosis vesicle biogenesis.

## IMPORTANCE

In Gram-negative bacteria, MV derived from the outer membrane have diverse functions in bacterial physiology and pathogenesis, and several factors regulating their production have been identified. Though Gram-positive bacteria and mycobacteria that lack an outer membrane also produce vesicles with described roles in pathogenesis, the mechanisms of MV biogenesis in these organisms remain poorly characterized. Defining mechanisms of MV biogenesis might yield significant insights into the importance of MV production during infection. In M. tuberculosis, only a single genetic element, *virR*, is known to regulate MV production. Our work reveals that the Pst/SenX3-RegX3 signal transduction system is a novel regulator of MV biogenesis that controls MV production by a mechanism that is independent of both VirR and activation of the specialized ESX-5 protein secretion system. Understanding which genes in the RegX3 regulon cause increased MV production might reveal novel molecular mechanisms of MV release.

## INTRODUCTION

Virtually all Gram-negative bacteria produce membrane vesicles (MV) by passive budding from the outer membrane (1). For many years, it was assumed that Gram-positive bacteria and mycobacteria would not release MV due to the difficulty of transporting membranous material through their complex outer cell walls. In the last decade, however, MV production has been described for many of these organisms, including the mycobacteria (2), although the mechanisms of MV release are not well understood (3).

Mycobacterium tuberculosis, the etiologic agent of tuberculosis in humans, produces MV derived from the inner membrane that modulate the host immune response (2, 4). MV contain glycolipids, including lipoarabinomannan (LAM), that inhibit CD4^+^ T cell activation (4) and lipoproteins, such as LpqH, that act in a TLR2-dependent manner to elicit the production of inflammatory cytokines and chemokines, including interleukin 1β (IL-1β), IL-6, IL-12, tumor necrosis factor alpha (TNF-α), and CXCL1 (2). LpqH can also regulate CD4^+^ T cell activation and macrophage major histocompatibility complex (MHC) class II expression (5, 6). It is unclear whether these MV-induced responses are beneficial or detrimental to bacterial survival. In mice pretreated with MV via the intratracheal route, these inflammatory responses impaired control of subsequent M. tuberculosis infection (2). However, when MV were administered systemically via subcutaneous injection, they induced a level of protection similar to that produced by the Mycobacterium bovis BCG vaccine (7). Furthermore, an M. tuberculosis mutant that produces more MV was hyper-inflammatory, inducing greater production of TNF-α and IL-6 from infected primary human macrophages, and this mutant was attenuated compared to wild-type (WT) M. tuberculosis (8). M. tuberculosis MV-null mutants might be used to conclusively determine the role of either MV-induced inflammation or MV-mediated inhibition of T cell function in pathogenesis, but such mutants have yet to be described.

MV production is an active and regulated process, since MV release can be induced by iron limitation (9), but the mechanisms that control MV biogenesis remain largely uncharacterized. The only genetic element currently known to affect M. tuberculosis MV production is *virR* (locus tag *rv0431*); disruption of *virR* resulted in increased MV release (8). VirR is a cytosolic protein that associates with the inner membrane and was proposed to act as part of a higher-order complex to regulate MV production through an unknown mechanism. Genetic screens in the model Gram-negative organism Escherichia coli revealed that numerous unique mutations cause increased MV release (10, 11), suggesting that *virR* is unlikely to be the only regulator of MV production in M. tuberculosis.

In addition to producing MV, M. tuberculosis interacts with the host via several protein secretion systems. Among these are five specialized type VII secretion systems, collectively referred to as the ESX systems (12). ESX-1, -3, and -5 each play an important role in pathogenesis. ESX-1 mediates the escape of M. tuberculosis from the phagosome and helps activate the inflammasome (13, 14). Furthermore, ESX-1 is required to trigger type I interferon responses, which are dependent on the cytosolic DNA sensor cyclic GMP-AMP synthase (cGAS) (14, 15). ESX-3 is essential for bacterial growth; it plays a role in mycobactin-mediated iron acquisition (16) but also has an iron-independent role in virulence (17). ESX-5 is important for inducing the inflammatory response and IL-1β production, as well as causing caspase-dependent cell death (18). These responses likely aid bacterial survival within the host (18). EccD_5_, the protein predicted to form the ESX-5 transmembrane channel, is required for full pathogenicity in macrophages and severe combined immunodeficiency (SCID) mice (19–21). We recently reported that ESX-5 activity is regulated by the Pst/SenX3-RegX3 system in response to extracellular inorganic phosphate (P_i_) availability (22).

The Pst (phosphate-specific transport) system is a high-affinity, low-velocity, ABC-type transport system. We have previously shown that the Pst system controls gene expression in response to extracellular P_i_ availability through an interaction with the SenX3-RegX3 two-component signal transduction system (23). This system, comprising a membrane-bound sensor histidine kinase (SenX3) and DNA binding response regulator (RegX3), is inhibited by the Pst system when P_i_ is abundant. When P_i_ is limiting, however, inhibition by the Pst system is relieved, leading to activation of SenX3-RegX3. Deletion of *pstA1*, which encodes a transmembrane component of the Pst system, causes constitutive activation of SenX3-RegX3 regardless of P_i_ availability (23). We demonstrated that in addition to controlling expression of genes involved in the P_i_ scavenging response, RegX3 directly binds and regulates transcription of *esx-5* genes (22, 23). The Δ*pstA1* mutant exhibits a RegX3-dependent increase in *esx-5* transcription and hyper-secretion of the ESX-5 substrates PPE41 and EsxN (22).

Here, we describe that in addition to affecting protein secretion via ESX-5, the Δ*pstA1* mutant hyper-secretes the lipoproteins LpqH and PstS1, which are released from M. tuberculosis in association with MV (2, 8, 24). The ESX-5 substrates EsxN and PPE41 have also previously been detected in association with MV by proteomic approaches (2, 24). We therefore hypothesized that the Pst/SenX3-RegX3 system regulates MV production and that MV release depends on the ESX-5 system. We show that while the Δ*pstA1* mutant does hyper-secrete MV in a RegX3-dependent manner, MV release occurs independently of ESX-5 activity. Furthermore, our data indicate that MV hyper-secretion from the Δ*pstA1* mutant is independent of VirR. Our data suggest that genes under the control of RegX3 impact MV release from Δ*pstA1* bacteria by a novel mechanism.

## RESULTS

### The Δ*pstA1* mutant hyper-secretes the lipoproteins LpqH and PstS1.

**FIG 1:**
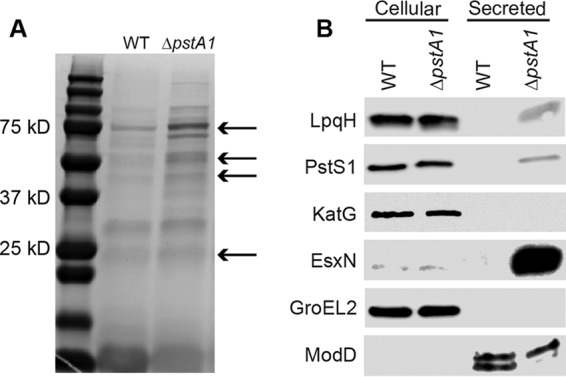
The Δ*pstA1* mutant hyper-secretes the lipoproteins LpqH and PstS1. Wild-type M. tuberculosis Erdman (WT) and Δ*pstA1* mutant cultures were grown for 5 days in Sauton’s complete medium without Tween 80. Cellular (10 µg) and secreted (5 µg) proteins were separated by SDS-PAGE. (A) Secreted proteins were stained with Imperial protein stain. Arrows indicate the bands selected for analysis by proteomic mass spectrometry. (B) Western blot analysis of proteins identified by mass spectrometry.

We previously demonstrated that ESX-5 secretion system activity is regulated in response to P_i_ availability by the Pst/SenX3-RegX3 signal transduction system (22). A Δ*pstA1* mutant, in which the RegX3 response regulator is constitutively activated, hyper-secretes the ESX-5 substrates EsxN and PPE41 under standard P_i_-replete culture conditions (22). We observed that the Δ*pstA1* mutant has a secreted protein profile distinct from that of the wild-type (WT) control (Fig. 1A). To preliminarily characterize these differences in protein secretion, we selected four protein bands at molecular weights of approximately 75, 50, 37, and 25 kDa that appeared to be hyper-secreted by the Δ*pstA1* mutant and subjected them to proteomic mass spectrometry (MS) analysis. Multiple proteins were identified in each band; the complete data are provided in Table S1 in the supplemental material.

10.1128/mBio.00778-18.5 TABLE S1  Proteomic mass spectrometry analysis of proteins hyper-secreted by the Δ*pstA1* mutant. Proteins analyzed by Western blotting are highlighted in yellow. Download TABLE S1, XLSX file, 0.7 MB. Copyright © 2018 White et al. 2018 White et al. This content is distributed under the terms of the Creative Commons Attribution 4.0 International license.

We obtained antibodies against several proteins identified by mass spectrometry (LpqH, PstS1, and KatG) and tested secretion of these proteins by Western blotting. We detected increased secretion of the lipoproteins LpqH and PstS1 by the Δ*pstA1* mutant compared to their levels in the WT control, though there was no change in the abundance of these proteins in the cellular fraction (Fig. 1B). As in our previous report, we observed increased secretion of the ESX-5 substrate EsxN from the Δ*pstA1* mutant compared to its level in the WT control. EsxN was undetectable in the secreted WT fraction when 5 µg of protein was loaded, but we previously reported that EsxN secretion is induced at least 8-fold in the Δ*pstA1* mutant (22). The catalase-peroxidase KatG was detected only in the cellular fraction of both strains, and as such, it was excluded from further analyses (Fig. 1B). GroEL2 served as a loading control for cell-associated protein and indicated that cellular lysis did not contaminate the secreted protein fraction. ModD, a protein secreted by the general Sec system, served as a control for equivalent loading of secreted proteins; we detected ModD as a doublet, consistent with its glycosylation (25). These data corroborated the mass spectrometry identification of LpqH and PstS1 as being hyper-secreted by the Δ*pstA1* mutant.

### The Δ*pstA1* mutant overproduces membrane vesicles containing the lipoproteins LpqH and PstS1.

**FIG 2:**
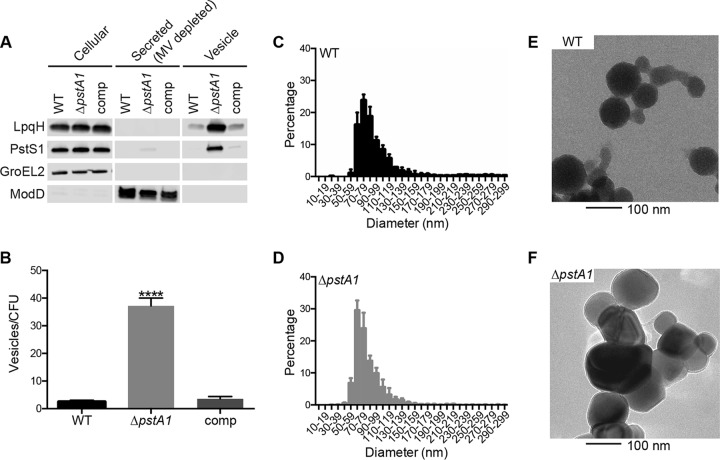
The Δ*pstA1* mutant hyper-secretes membrane vesicles containing LpqH and PstS1. Cultures of wild-type M. tuberculosis Erdman (WT), the Δ*pstA1* mutant, and the Δ*pstA1* pMV*pstA1* complemented strain (comp) were grown for 5 days in Sauton’s complete medium without Tween 80, and membrane vesicles were isolated. (A) Cellular proteins (5 µg), secreted proteins depleted of MV (5 µg), and MV suspension (20 µl) were analyzed by Western blotting to detect the indicated proteins. (B to D) Nanoparticle tracking analysis of culture supernatants. (B) Numbers of particles per milliliter were normalized to numbers of CFU per milliliter calculated from a control culture of each strain grown in Sauton’s complete medium with Tween 80. Data are means ± standard deviations for three independent cultures. ****, *P* < 0.0001. (C and D) Distribution of particle sizes from the WT and Δ*pstA1* strains, binned in 10-nm increments. (E and F) Transmission electron micrographs of membrane vesicles purified and concentrated from culture supernatants of WT and Δ*pstA1* cultures.

The LpqH and PstS1 lipoproteins were previously described to be associated with M. tuberculosis MV (2, 8, 24). Based on their lipid composition, mycobacterial MV are derived from the inner membrane and contain lipoproteins and other protein cargo (2, 24). Since both LpqH and PstS1 were hyper-secreted by the Δ*pstA1* mutant, we sought to determine whether these proteins were MV associated. We isolated MV from culture filtrates by ultracentrifugation and performed Western blotting experiments to analyze the distribution of LpqH and PstS1. Extracellular LpqH and PstS1 were localized exclusively to the MV fraction, and both proteins were more abundant in MV released from the Δ*pstA1* mutant than in the WT control (Fig. 2A). Complementation of the Δ*pstA1* mutation restored the MV-associated release of LpqH and PstS1 to levels comparable to those in the WT control.

To quantify MV release, we conducted nanoparticle-tracking analysis (NTA) on culture supernatants collected from WT, Δ*pstA1*, and Δ*pstA1* pMV*pstA1* bacteria using a NanoSight instrument. We observed a statistically significant 15-fold increase in MV release from Δ*pstA1* bacteria compared to that in WT bacteria but no difference in MV release between the WT and Δ*pstA1* pMV*pstA1* strains (Fig. 2B). The accompanying size analysis of MV isolated from WT and Δ*pstA1* bacteria revealed that both strains produced particles that fell within the 40- to 250-nm-diameter range previously observed for M. tuberculosis MV (2) (Fig. 2C and D). To further confirm these results, purified MV from these strains were imaged via transmission electron microscopy (TEM). We observed vesicular structures with the predicted size of MV from both WT and Δ*pstA1* mutant bacteria (Fig. 2E and F). Taken together, these data support the finding that the Δ*pstA1* mutant releases an increased number of MV, and these vesicles are enriched for both the LpqH and PstS1 lipoproteins.

### Overproduction of membrane vesicles by the Δ*pstA1* mutant requires RegX3.

**FIG 3:**
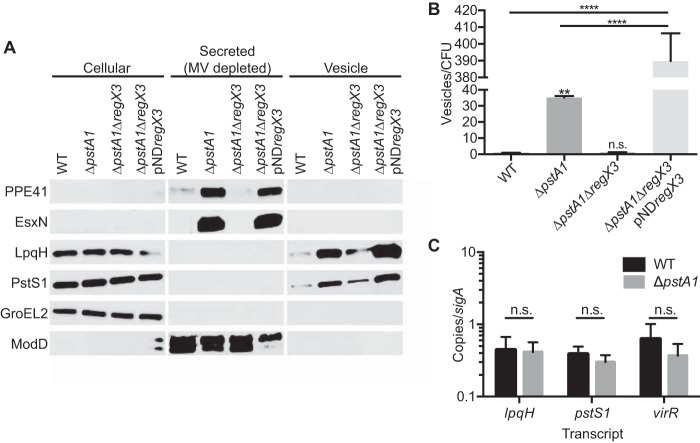
Increased release of membrane vesicles from the Δ*pstA1* mutant requires RegX3. Wild-type M. tuberculosis Erdman (WT) and the Δ*pstA1*, Δ*pstA1* Δ*regX3*, and Δ*pstA1* Δ*regX3* pND*regX3* strains were grown for 5 days in Sauton’s complete medium without Tween 80. (A) Cellular proteins (5 µg), secreted proteins depleted of MV (5 µg), and MV suspension (20 µl) were separated and analyzed by Western blotting to detect the indicated proteins. (B) Nanoparticle tracking analyses of culture supernatants. The results were normalized to numbers of CFU per milliliter determined from a control culture grown in Sauton’s medium with Tween 80 and plated on 7H10 medium. Data are means ± standard deviations for three independent cultures. **, *P* < 0.01; ****, *P* < 0.0001. n.s., the difference was not significant. (C) The transcript abundance of *lpqH*, *pstS1*, and *virR* relative to that of *sigA* were determined by quantitative RT-PCR for the WT and Δ*pstA1* strains grown to mid-logarithmic phase in 7H9 complete medium. Results are the means ± standard deviations for three independent experiments.

Many of the phenotypes that we have previously reported for the Δ*pstA1* mutant are dependent on RegX3 (22, 23, 26, 27). To determine whether constitutive activation of RegX3 also contributes to overproduction of MV by the Δ*pstA1* mutant, we performed additional Western blotting and NTA on the Δ*pstA1* Δ*regX3* and Δ*pstA1* Δ*regX3* pND*regX3* strains. Hyper-secretion of the ESX-5 substrates EsxN and PPE41 was abolished in the Δ*pstA1* Δ*regX3* mutant (Fig. 3A), as previously described (22). EsxN and PPE41 were detected only in the secreted protein fraction despite previous reports from proteomic analyses that these proteins are MV associated (2, 24). This is likely due to the lower sensitivity of Western blots than of proteomic mass spectrometry. Both LpqH and PstS1 were detected in the vesicle fraction from the Δ*pstA1* Δ*regX3* mutant but at reduced levels compared to those in the Δ*pstA1* strain (Fig. 3A). In all cases, complementation of the Δ*regX3* deletion with pND*regX3* restored hyper-secretion (Fig. 3A).

We again used NTA to quantify MV release from the *regX3* deletion mutant and complemented strains. Size analysis revealed that all strains produced MV of the appropriate diameter, with the majority clustering between 50 and 100 nm (Fig. S1). As suggested by the reduced abundance of LpqH and PstS1 in the vesicle fraction from the Δ*pstA1* Δ*regX3* strain, NTA revealed that vesicle release from the Δ*pstA1* Δ*regX3* mutant returned to WT levels (Fig. 3B). When the *regX3* deletion was complemented, we observed a significant increase in MV release of more than 10-fold compared to MV release in the Δ*pstA1* strain. We hypothesize that this increase is due to overexpression of *regX3* from the complementation vector, as previously reported (23). We observed no significant difference in abundance of the *lpqH* or *pstS1* transcripts between the Δ*pstA1* mutant and WT bacteria (Fig. 3C), consistent with no apparent change in LpqH or PstS1 production in the cellular protein fraction (Fig. 3A). These results demonstrate that increased release of LpqH and PstS1 associated with MV is not due to changes in their expression. Taken together, these results indicate that some other factor, or factors, regulated by RegX3 influence MV production.

10.1128/mBio.00778-18.1 FIG S1  The Δ*pstA1* Δ*regX3* and Δ*pstA1* Δ*regX3* pND*regX3* strains produce membrane vesicles. The Δ*pstA1* Δ*regX3* and Δ*pstA1* Δ*regX3* pND*regX3* strains were grown for 5 days in Sauton’s complete medium without Tween 80. Nanoparticle tracking analyses were performed on culture supernatants, and the resulting particle sizes were binned in 10-nm increments. Download FIG S1, PDF file, 0.5 MB. Copyright © 2018 White et al. 2018 White et al. This content is distributed under the terms of the Creative Commons Attribution 4.0 International license.

### Increased vesicle production by Δ*pstA1* bacteria is not dependent on VirR.

**FIG 4:**
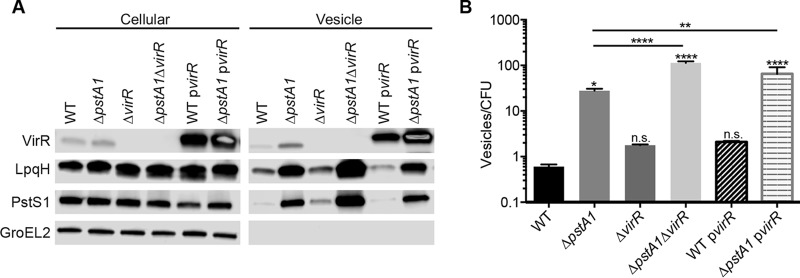
Increased vesicle release from the Δ*pstA1* mutant is independent of VirR. Wild-type M. tuberculosis Erdman (WT) and the Δ*pstA1*, Δ*virR*, Δ*pstA1* Δ*virR*, WT p*virR*, and Δ*pstA1* p*virR* strains were grown for 5 days in Sauton’s complete medium without Tween 80. (A) Cellular proteins (5 µg) and MV suspension (20 µl) were analyzed by Western blotting to detect the indicated proteins. (B) Nanoparticle tracking analysis of culture supernatants. Numbers of particles per milliliter were normalized to numbers of CFU per milliliter determined from a control culture grown in Sauton’s medium with Tween 80 and plated on 7H10 medium. Data are means ± standard deviations for three independent cultures. The results of all statistical analyses are compared to WT levels unless otherwise indicated. *, *P* < 0.05; **, *P* < 0.01; ****, *P* < 0.0001.

*virR* was previously implicated as a regulator of MV production; transposon disruption of *virR* led to increased MV biogenesis (8). We analyzed the transcription of *virR* to determine whether changes in *virR* expression contribute to increased MV release. While the difference was not statistically significant, we observed a 1.7-fold decrease in the *virR* transcript level in the Δ*pstA1* mutant compared to that in the WT control (Fig. 3C). We investigated whether decreased *virR* transcript abundance could account for increased MV release from the Δ*pstA1* strain. Western blotting revealed similar levels of VirR production in the cellular fractions of WT and Δ*pstA1* bacteria, suggesting that the modest differences in *virR* transcript abundance do not affect VirR protein production (Fig. 4A). VirR associates with MV and is more abundant in MV from the Δ*pstA1* strain, consistent with enhanced MV release (Fig. 4A).

We took two approaches to determine whether MV production by the Δ*pstA1* mutant depends on *virR*. We overexpressed FLAG-tagged VirR (p*virR*) (8) to restore *virR* expression. Western blots of the WT p*virR* and Δ*pstA1* p*virR* strains revealed a dramatic overproduction of VirR (Fig. 4A). We also constructed a deletion in *virR*, which we predicted would not affect MV production by the Δ*pstA1* mutant if both PstA1 and VirR act in the same MV biogenesis pathway. To avoid polarity on adjacent essential genes, we deleted amino acid residues 85 to 103 of VirR, which correspond to the core globular structure of the protein (8). Western blotting confirmed loss of VirR production in both the Δ*virR* and the Δ*pstA1* Δ*virR* strains (Fig. 4A).

The overexpression of *virR* did not dramatically alter LpqH or PstS1 abundance in the MV fraction. However, VirR was much more abundant in MV from both the WT p*virR* and the Δ*pstA1* p*virR* strains (Fig. 4A), indicating that VirR may become overrepresented in MV when it is overproduced. Both VirR overexpression strains had slightly increased MV production compared to that of the corresponding parental controls, but this was statistically significant only for the Δ*pstA1* p*virR* strain (Fig. 4B). Notably, Δ*pstA1* p*virR* bacteria still produced more MV than WT bacteria, demonstrating that *virR* overexpression does not rescue the MV hyper-secretion phenotype of the Δ*pstA1* strain (Fig. 4B).

The Δ*virR* strain had slightly more LpqH and PstS1 in the MV fraction than the WT control (Fig. 4A), suggesting increased MV production as previously reported (8). Both LpqH and PstS1 were also more abundant in the MV fraction from the Δ*pstA1* Δ*virR* mutant than in either single mutant or WT bacteria (Fig. 4A). NTA confirmed that the Δ*virR* strain produced 3-fold-more MV than the WT strain, although this increase was not statistically significant (Fig. 4B). NTA also confirmed that the Δ*pstA1* Δ*virR* strain produced 4-fold-more MV than the Δ*pstA1* mutant and 63-fold-more MV than the Δ*virR* mutant, both significant increases (Fig. 4B). Taken together, these results indicate that overproduction of MV by the Δ*pstA1* strain is independent of VirR. Furthermore, the mechanisms driving increased MV production in the Δ*pstA1* and Δ*virR* strains appear to synergize with each other, as a strain harboring deletions of both of these genes produced significantly more MV than either single mutant.

### Construction of conditional *eccD*_*5*_ depletion mutants.

We previously demonstrated that the Δ*pstA1* mutant exhibits increased ESX-5 activity that is dependent on the RegX3 response regulator (22). Furthermore, others have reported detection of ESX-5 substrates within MV (2, 24). We therefore sought to determine whether increased MV production by the Δ*pstA1* mutant was due to increased ESX-5 activity. We attempted to delete *eccD*_*5*_, which encodes a core component of the ESX-5 secretion system that is predicted to form an inner membrane pore through which substrates are secreted (21, 28). We constructed a Δ*eccD*_*5*_ allelic-exchange vector and introduced it into both the WT Erdman and Δ*pstA1* mutant strain backgrounds. After obtaining cointegrates with the plasmid integrated at the *eccD*_*5*_ locus, we screened sucrose-resistant colonies for the Δ*eccD*_*5*_ deletion by PCR. All the colonies screened (130 with the WT background, 169 with the Δ*pstA1* background) retained the WT *eccD*_*5*_ allele, suggesting that *eccD*_*5*_ is essential for the replication of M. tuberculosis in the standard Middlebrook 7H9 and 7H10 media that we used.

To create strains for conditional expression of *eccD*_*5*_, we first introduced a plasmid (pTIC-*eccD*_*5*_) in which *eccD*_*5*_ transcription is under the control of a tetracycline-inducible (Tet-ON) promoter. We then attempted to delete the chromosomal copy of *eccD*_*5*_ in the WT pTIC-*eccD*_*5*_ and Δ*pstA1* pTIC-*eccD*_*5*_ strains. All strain construction steps were conducted in the presence of anhydrotetracycline (ATc) to stimulate *eccD*_*5*_ transcription from pTIC-*eccD*_*5*_. We screened 8 and 16 sucrose-resistant, hygromycin (Hyg)-sensitive isolates in the WT and Δ*pstA1* mutant backgrounds, respectively. Among these isolates, we identified 2 Δ*eccD*_*5*_ pTIC-*eccD*_*5*_ mutants and 2 Δ*pstA1* Δ*eccD*_*5*_ pTIC-*eccD*_*5*_ mutants that we herein refer to as *eccD*_*5*_ Tet-ON and Δ*pstA1 eccD*_*5*_ Tet-ON, respectively. These data support the contention that *eccD*_*5*_ is essential for the replication of M. tuberculosis under standard *in vitro* culture conditions.

We analyzed the growth of the *eccD*_*5*_ Tet-ON and Δ*pstA1 eccD*_*5*_ Tet-ON strains compared to that of the parental controls in liquid medium and on agar plates. Both *eccD*_*5*_ Tet-ON strains had a growth defect in liquid medium compared to the growth of the respective parental control even in the presence of ATc to induce *eccD*_*5*_ transcription (Fig. S2A and B). The *eccD*_*5*_ Tet-ON strains also produced smaller colonies on agar plates regardless of ATc treatment (data not shown). Although *eccD*_*5*_ transcription was induced in the *eccD*_*5*_ Tet-ON strains in the presence of ATc, there was still detectable transcript in no-ATc controls (Fig. S2C), and we observed no difference in EccD_5_ protein abundance between treatment conditions (Fig. S2D). Additionally, PPE41 was secreted even in the absence of ATc (Fig. S2D). Overall, our data suggest that leaky expression from the Tet-inducible promoter in the absence of ATc is sufficient to support the normal activity of the ESX-5 secretion system in the *eccD*_*5*_ Tet-ON strains.

10.1128/mBio.00778-18.2 FIG S2  Residual *eccD*_*5*_ expression in uninduced *eccD*_*5*_ Tet-ON strains is sufficient for protein secretion through ESX-5. Kanamycin (15 µg/ml) was added to all *eccD*_*5*_ Tet-ON cultures to maintain plasmid integration. (A and B) Wild-type M. tuberculosis Erdman (WT) and the Δ*pstA1*, *eccD*_*5*_ Tet-ON, and Δ*pstA1 eccD*_*5*_ Tet-ON strains were inoculated in 7H9 complete medium at an OD_600_ of 0.05 and grown at 37°C with aeration. Anhydrotetracycline hydrochloride (ATc; 50 ng/ml) was added every 3 days to the indicated cultures. Growth was monitored by daily OD_600_ measurements. (C) The transcript abundance of *eccD*_*5*_ relative to that of *sigA* was determined by quantitative RT-PCR for the WT, Δ*pstA1*, *eccD*_*5*_ Tet-ON, and Δ*pstA1 eccD*_*5*_ Tet-ON strains grown to mid-logarithmic phase in 7H9 complete medium with and without ATc. (D) The WT, Δ*pstA1*, *eccD*_*5*_ Tet-ON, and Δ*pstA1 eccD*_*5*_ Tet-ON strains were grown for 5 days in Sauton’s complete medium without Tween 80. ATc (50 ng/ml) was added to the indicated cultures to induce *eccD*_*5*_. Equivalent amounts of cellular (5 µg) and secreted (5 µg) proteins were analyzed by Western blotting to detect the indicated proteins. Download FIG S2, PDF file, 0.8 MB. Copyright © 2018 White et al. 2018 White et al. This content is distributed under the terms of the Creative Commons Attribution 4.0 International license.

We therefore turned to construction of strains harboring tetracycline-repressible *eccD*_*5*_ (*eccD*_*5*_ Tet-OFF). We cloned *eccD*_*5*_ under the control of the *tetO*-4C5G operator (29) on a vector that integrates at the same site as pTIC10a. This pGMCH *eccD*_*5*_ Tet-OFF vector was introduced into the *eccD*_*5*_ Tet-ON strains, and integrants in which pGMCH *eccD*_*5*_ Tet-OFF replaced pTIC-*eccD*_*5*_ by a plasmid swap were selected on 7H10 containing hygromycin. We recovered over 15-fold-more colonies from electroporation with pGMCH *eccD*_*5*_ Tet-OFF than with an empty hygromycin resistance (Hyg^r^) plasmid control. The majority of pGMCH *eccD*_*5*_ Tet-OFF transformants were hygromycin resistant and kanamycin (Kan) sensitive, while isolates transformed with the empty Hyg^r^ plasmid were resistant to both hygromycin and kanamycin, indicating that they retained the pTIC-*eccD*_*5*_ plasmid. These data provide further evidence that *eccD*_*5*_ is essential for M. tuberculosis replication *in vitro*. Strains in which the pGMCH *eccD*_*5*_ Tet-OFF plasmid replaced pTIC-*eccD*_*5*_ were confirmed by PCR and designated *eccD*_*5*_ Tet-OFF and Δ*pstA1 eccD*_*5*_ Tet-OFF. We also attempted construction of dual-control strains in which both *eccD*_*5*_ transcriptional repression and EccD_5_ protein degradation would occur upon addition of ATc due to the addition of a DAS+4 degradation tag on EccD_5_ (29). However, attempts to introduce the plasmid encoding DAS-tagged EccD_5_ by plasmid swap produced <10 colonies, all of which were resistant to both hygromycin and kanamycin, suggesting that EccD_5_-DAS+4 is nonfunctional.

**FIG 5:**
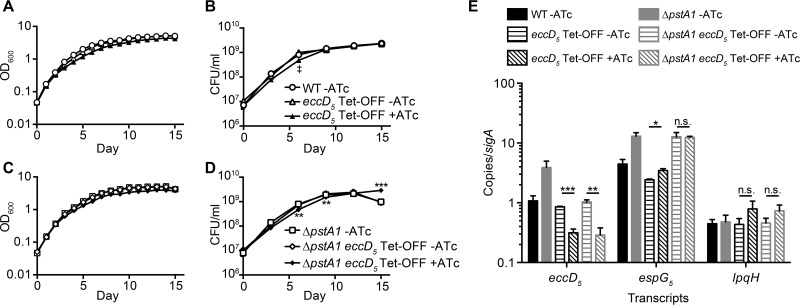
Incomplete repression of *eccD*_*5*_ transcription has moderate effects on growth. (A to D) Wild-type M. tuberculosis Erdman (WT) and the Δ*pstA1*, *eccD*_*5*_ Tet-OFF, and Δ*pstA1 eccD*_*5*_ Tet-OFF strains were inoculated in 7H9 complete medium at an OD_600_ of 0.05 and grown at 37°C with aeration. Anhydrotetracycline hydrochloride (ATc; 100 ng/ml) was added at day 0 and day 7 as indicated. Growth was monitored by daily OD_600_ measurements (A and C) and by plating serially diluted cultures on 7H10 medium to determine numbers of viable CFU per milliliter on days 0, 3, 6, 9, 12, and 15 (B and D). The key in panel B applies to panels A and B; the key in panel D applies to panels C and D. **, *P* < 0.01; ***, *P* < 0.001; ‡, *P* = 0.057. (E) The transcript abundances of *eccD*_*5*_, *espG*_*5*_, and *lpqH* relative to that of *sigA* were determined by quantitative RT-PCR for the WT, Δ*pstA1*, *eccD*_*5*_ Tet-OFF, and Δ*pstA1 eccD*_*5*_ Tet-OFF strains grown to mid-logarithmic phase in 7H9 complete medium with and without ATc. Results are the means ± standard deviations for three independent experiments. *, *P* = 0.0167, **; *P* = 0.0042; ***, *P* = 0.0005.

**FIG 6:**
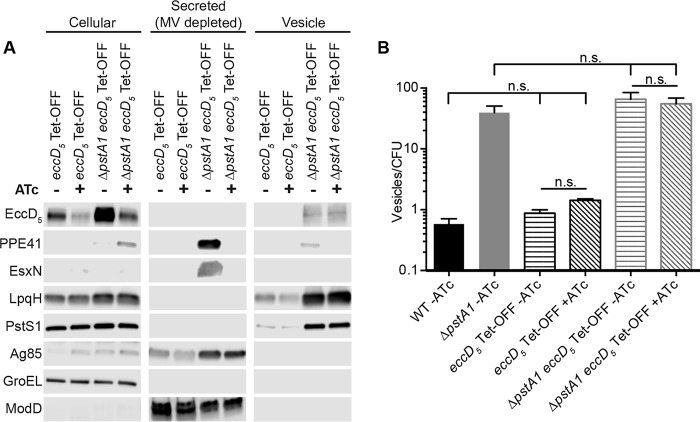
Depletion of EccD_5_ prevents ESX-5 secretion but does not affect membrane vesicle production. The *eccD*_*5*_ Tet-OFF and Δ*pstA1 eccD*_*5*_ Tet-OFF mutants were grown for 5 days in Sauton’s complete medium without Tween 80. Anhydrotetracycline hydrochloride (ATc; 100 ng/ml) was added as indicated to repress *eccD*_*5*_. (A) Cellular proteins (5 µg), secreted proteins depleted of MV (5 µg), and MV suspension (20 µl) were analyzed by Western blotting to detect the indicated proteins. (B) Nanoparticle tracking analysis of culture supernatants. Numbers of particles per milliliter were normalized to numbers of CFU per milliliter determined from a control culture of each strain grown with Tween 80 and plated on 7H10 complete medium. The data are means ± standard deviations for three independent cultures.

The *eccD*_*5*_ Tet-OFF strains in both the WT and Δ*pstA1* backgrounds had statistically significant growth defects (*P* < 0.05) in liquid medium containing ATc beginning at day 3 compared to the growth of the no-drug controls (Fig. 5A and C). These defects were maintained throughout the incubation period until day 15, when they dropped just below the level of statistical significance. Similarly, we recovered fewer viable CFU from cultures of both *eccD*_*5*_ Tet-OFF strains at days 3 to 12 when ATc was added (Fig. 5B and D). These differences did not quite achieve statistical significance for the *eccD*_*5*_ Tet-OFF strain (Fig. 5B). Addition of ATc significantly reduced the growth of the Δ*pstA1 eccD*_*5*_ Tet-OFF strain at days 6 and 9, but by day 15, we observed a significant increase in the number of viable CFU recovered from these cultures (Fig. 5D). Both *eccD*_*5*_ Tet-OFF strains produced smaller colonies when grown on 7H10 medium with ATc (Fig. S3). Importantly, the addition of ATc did not affect the growth kinetics or colony morphology of the WT or Δ*pstA1* parent strain (Fig. S3 and S4). Quantitative reverse transcription-PCR (qRT-PCR) confirmed that addition of ATc to the *eccD*_*5*_ Tet-OFF and Δ*pstA1 eccD*_*5*_ Tet-OFF strains resulted in significant 2.7-fold and 3.6-fold repression of *eccD*_*5*_, respectively (Fig. 5E). This repression did not dramatically alter the expression of other genes within the ESX-5 locus, such as *espG*_*5*_, or the expression of *lpqH* itself (Fig. 5E). To confirm that reduced *eccD*_*5*_ transcription altered EccD_5_ protein production and ESX-5 secretion system activity, we performed Western blotting. EccD_5_ production was reduced 2.4-fold and 3.5-fold in the *eccD*_*5*_ Tet-OFF and Δ*pstA1 eccD*_*5*_ Tet-OFF strains, respectively, in the presence of ATc (Fig. 6A). These results suggest that *eccD*_*5*_ expression is not fully repressed by ATc in the Tet-OFF strains and that sufficient EccD_5_ remains to support growth, even when the cultures contain ATc.

10.1128/mBio.00778-18.3 FIG S3  Repression of *eccD*_*5*_ results in small-colony morphology. Wild-type M. tuberculosis Erdman (WT) and the Δ*pstA1*, *eccD*_*5*_ Tet-OFF, and Δ*pstA1 eccD*_*5*_ Tet-OFF strains were inoculated in 7H9 complete medium at an OD_600_ of 0.05. Cultures were serially diluted, and 10 µl of the indicated dilutions were spot plated on 7H10 complete medium with or without ATc (100 ng/ml). Download FIG S3, PDF file, 10.4 MB. Copyright © 2018 White et al. 2018 White et al. This content is distributed under the terms of the Creative Commons Attribution 4.0 International license.

10.1128/mBio.00778-18.4 FIG S4  ATc does not affect the growth of WT or Δ*pstA1* bacteria. Wild-type M. tuberculosis Erdman (WT) and the Δ*pstA1* strain were inoculated in 7H9 complete medium at an OD_600_ of 0.05 and grown at 37°C with aeration. Anhydrotetracycline hydrochloride (ATc; 100 ng/ml) was added at day 0 and day 7 as indicated. Growth was monitored by daily OD_600_ measurements (A and C) and by plating serially diluted cultures on 7H10 medium to determine numbers of viable CFU per milliliter on days 0, 3, 6, 9, 12, and 15 (B and D). Download FIG S4, PDF file, 0.5 MB. Copyright © 2018 White et al. 2018 White et al. This content is distributed under the terms of the Creative Commons Attribution 4.0 International license.

We observed typical hyper-secretion of both EsxN and PPE41 from the Δ*pstA1 eccD*_*5*_ Tet-OFF strain in the absence of ATc, but secretion of these proteins was abrogated when ATc was added (Fig. 6A). Treatment of the Δ*pstA1 eccD*_*5*_ Tet-OFF strain with ATc also caused an increase in PPE41 associated with the cellular fraction (Fig. 6A), indicating that PPE41 becomes trapped within the cell when ESX-5 secretion is prevented. We were able to faintly detect PPE41 only in the MV fraction of the untreated Δ*pstA1 eccD*_*5*_ Tet-OFF strain, further supporting our finding that the majority of secreted EsxN and PPE41 is not MV associated. Other secreted proteins, such as the antigen 85 (Ag85) complex and ModD, were not affected by the repression of *eccD*_*5*_ (Fig. 6A). Taken together, these analyses confirm that the Tet-OFF strains function as expected and demonstrate that repression of *eccD*_*5*_ results in reduced secretion through ESX-5.

### The ESX-5 secretion system is not required for membrane vesicle release.

We predicted that repression of *eccD*_*5*_ transcription in the Δ*pstA1 eccD*_*5*_ Tet-OFF strain would prevent MV hyper-secretion. MV were isolated from *eccD*_*5*_ Tet-OFF and Δ*pstA1 eccD*_*5*_ Tet-OFF strains as described above but with the addition of ATc to the indicated cultures during the final growth phase. Western blots for LpqH and PstS1 demonstrated that MV were hyper-secreted from the Δ*pstA1 eccD*_*5*_ Tet-OFF strain in similar amounts whether or not ATc was added to the cultures (Fig. 6A). NTA confirmed that there was no significant difference in the numbers of MV released between samples with and without ATc treatment or between the Tet-OFF strains and the corresponding parental control (Fig. 6B). These findings refute our initial hypothesis and instead indicate that ESX-5 secretion system activity is not required for MV release from M. tuberculosis.

## DISCUSSION

M. tuberculosis produces MV, but the molecular mechanisms and regulatory processes controlling their release are poorly characterized. We demonstrate that the Δ*pstA1* mutant hyper-secretes MV containing the lipoproteins LpqH and PstS1 in a RegX3-dependent manner. Because the Δ*pstA1* mutant also exhibits a RegX3-dependent increase in the activity of the ESX-5 secretion system and ESX-5 substrates had previously been identified within MV, we initially hypothesized that MV release and ESX-5 activity were connected. However, the overproduction of MV from the Δ*pstA1 eccD*_*5*_ Tet-OFF strain was not affected by repressing ESX-5 secretion. Our data demonstrate that the Pst/SenX3-RegX3 system regulates the production of MV independently of ESX-5 activity.

Our data suggest that one or more factors regulated by RegX3 causes increased MV release from the Δ*pstA1* mutant. Previous work implicated *virR* as a regulator of MV production (8), but we observed only modest, insignificant changes in *virR* expression in Δ*pstA1* bacteria compared to its expression in the WT. Furthermore, deletion of *virR* in the Δ*pstA1* background resulted in an even greater increase in MV production, suggesting that these genes promote MV production through separate mechanisms that are capable of synergizing with each other. Overexpression of *virR* in the Δ*pstA1* background also resulted in a slight increase in MV production, providing further support for this hypothesis and suggesting that dramatic overproduction of VirR may also influence MV production. Together, these findings indicate that MV production is mediated by a novel mechanism in Δ*pstA1* bacteria. Over 60 genes are dysregulated in the Δ*pstA1* mutant in a RegX3-dependent manner (23), and any one or more of these genes may be involved in MV release. Our future studies will seek to identify the basis for increased MV release by the Δ*pstA1* mutant to provide mechanistic insight into MV biogenesis.

By carefully examining the RegX3 regulon, we developed several hypotheses to explain increased MV production from the Δ*pstA1* mutant. Several highly upregulated genes in the Δ*pstA1* mutant are involved, or predicted to be involved, in the production of lipoproteins and glycolipids. One of the most upregulated genes, *lppF*, was overexpressed 20-fold in the Δ*pstA1* mutant and is predicted to encode a lipoprotein (23). While LppF has not been detected in association with MV, several other lipoproteins, including LpqH, LppX, LprA, LprG, and LprF, are highly abundant (24). Additionally, *rv0557* (*mgtA*) transcript levels are almost 14-fold higher in Δ*pstA1* bacteria (23). The Corynebacterium glutamicum ortholog of this gene is required for production of a novel glycolipid, 1,2-di-*O*-C_16_/C_18:1_-(α-d-mannopyranosyl)-(1→4)-(α-d-glucopyranosyluronic acid)-(1→3)-glycerol (ManGlcAGroAc_2_), that contributes to the lipomannan (LM) pool (30). In M. tuberculosis, inactivation of *rv0557* results in reduced cell wall LAM and LM (31). As both LAM and LM are incorporated in MV (2, 4), it is possible that changes in the production of these lipoglycans influence MV release. Two genes encoding putative acyltransferases (*rv3027c* and *rv3026c*) are also upregulated in the Δ*pstA1* mutant 10- and 7-fold, respectively (23). These genes may contribute to increased lipid production, resulting in more MV biogenesis. Finally, *rv1491c* is also upregulated 3-fold in the Δ*pstA1* mutant (23) and encodes a DedA family protein. In E. coli, deletion of genes encoding DedA family proteins causes cell division defects and altered membrane phospholipid composition (32). We hypothesize that Rv1491c has similar roles in M. tuberculosis membrane biogenesis and thus influences MV production. Additionally, Rv1491c is encoded near Rv1488, a putative membrane protein that interacts with VirR (8). It is possible that both Rv1491c and Rv1488 play roles in MV biogenesis. We intend to explore these hypotheses as part of our future studies.

We initially hypothesized that ESX-5 would be involved in MV release based in part on prior detection of the ESX-5 substrates PPE41 and EsxN in MV by mass spectrometry (2, 24), yet we were generally unable to detect these proteins in MV by Western blotting. We observed the majority of PPE41 and EsxN only in the secreted protein fraction and confirmed that their secretion requires EccD_5_. Our results indicate that PPE41 and EsxN are secreted primarily as soluble protein via ESX-5.

Our data also suggest that the ESX-5 core component EccD_5_ is essential for M. tuberculosis viability. We were unable to delete *eccD*_*5*_ unless a complementing copy of the gene was provided in *trans*, and we also could not efficiently replace the complementing copy of *eccD*_*5*_ with an empty vector. Similarly, both *eccB*_*5*_ and *eccC*_*5*_, which encode two additional core components of ESX-5, could not be deleted without a complementing copy of the appropriate gene provided in *trans*, suggesting that they are also essential for M. tuberculosis viability (33). Conserved ESX-5 components *eccC*_*5*_ and *mycP*_*5*_ also appear to be essential for the growth of the mycobacterial species M. marinum and M. bovis BCG (34). Others have reported deletion (20) or disruption (35) of *eccD*_*5*_, in contrast to our data. It is possible that these mutants were still viable due to compensatory mutations. Indeed, *eccC*_*5*_ could be deleted in M. marinum mutants deficient for production of the virulence lipid phthiocerol dimycocerosate (PDIM) (34), and spontaneous loss of PDIM production by M. tuberculosis has previously been reported (36). Overall, our data support a growing body of literature suggesting that ESX-5 secretion in general and the EccD_5_ core component in particular are essential for mycobacterial growth *in vitro*.

To circumvent the essentiality of EccD_5_, we generated conditional *eccD*_*5*_ Tet-OFF strains in both the WT and Δ*pstA1* backgrounds. Addition of ATc to these strains repressed *eccD*_*5*_ transcription but caused only moderate growth defects either in liquid medium or on solid agar. EccD_5_ production was also decreased in both *eccD*_*5*_ Tet-OFF strains when ATc was added, although protein was still detectable by Western blotting. We hypothesize that the residual EccD_5_ produced by these strains was sufficient to support some growth. Nevertheless, depletion of EccD_5_ led to decreased ESX-5 activity, as indicated by reduced secretion of PPE41 and EsxN. Collectively, these data indicate that native EccD_5_ is necessary for M. tuberculosis viability and that lower *eccD*_*5*_ expression results in decreased growth and reduced protein secretion via ESX-5. Future work will use these *eccD*_*5*_ Tet-OFF strains to identify additional secreted substrates of the ESX-5 system and to investigate the role that ESX-5 secretion plays in pathogenesis.

## MATERIALS AND METHODS

### Strains and culture conditions.

M. tuberculosis Erdman and Δ*pstA1*, Δ*pstA1* pMV*pstA1*, Δ*pstA1* Δ*regX3*, and Δ*pstA1* Δ*regX3* pND-*regX3* strains were previously described (23). Construction of the Δ*virR*, Δ*pstA1* Δ*virR*, *virR* overexpression, *eccD*_*5*_ Tet-OFF, and Δ*pstA1 eccD*_*5*_ Tet-OFF strains are described below. Bacteria were routinely cultured in Middlebrook 7H9 liquid medium (Difco) supplemented with 10% albumin-dextrose-saline (ADS), 0.5% glycerol, and 0.1% Tween 80 or on Middlebrook 7H10 agar medium (Difco) supplemented with oleic acid-albumin-dextrose-catalase (OADC; BD Biosciences) and 0.5% glycerol. Strains containing the p*virR* plasmid were grown in the presence of 50 µg/ml hygromycin B (Sigma). Frozen stocks were prepared by growing cultures to mid-logarithmic phase, adding glycerol to a 15% final concentration, and aliquoting for storage at −80°C.

### Proteomic analysis.

M. tuberculosis WT and Δ*pstA1* bacteria were grown in Sauton’s medium without Tween 80, and the secreted protein fractions were isolated as previously described (22). Total secreted proteins (10 µg) were separated by sodium dodecyl sulfate-polyacrylamide gel electrophoresis (SDS-PAGE). The gel was fixed for 30 min in 40% ethanol-10% acetic acid and then stained with Imperial protein stain (Thermo Scientific) and destained in water. Bands of interest from three SDS-PAGE gel lanes of the Δ*pstA1* mutant corresponding to 75, 50, 37, and 25 kDa were excised from the gel, pooled, cut into ~2- by 2-mm cubes, and processed by in-gel trypsin digestion. Gel slices were washed twice for 15 min at room temperature in wash buffer (1:1 acetonitrile-100 mM NH_4_HCO_3_) and then treated with 100% acetonitrile until the pieces shrank and turned white and semiopaque. Proteins were reduced with 10 mM dithiothreitol (DTT) in 10 mM NH_4_HCO_3_ for 1 h at 56°C and then alkylated by treatment with 55 mM iodoacetamide in 100 mM NH_4_HCO_3_ for 30 min at room temperature in the dark. Gel slices were washed twice with wash buffer and then washed briefly with 100% acetonitrile before rehydration in digestion buffer (50 mM NH_4_HCO_3_, 5 mM CaCl_2_, 12.5 ng/µl trypsin) at 4°C for 15 min, followed by overnight incubation in 50 mM NH_4_HCO_3_, 5 mM CaCl_2_ at 37°C. Peptides were extracted from the gel slices with 50% acetonitrile, 0.3% formic acid for 15 min and then with 80% acetonitrile, 0.3% formic acid for 15 min. Peptides were incubated at −80°C for 30 min, dried in a speed vac, and stored at −80°C prior to analysis by liquid chromatography-tandem mass spectrometry (LC/MS-MS) on an LTQ Orbitrap Velos mass spectrometer (Thermo, Fisher). The MS-MS data were compared with sequences in an M. tuberculosis proteomic database using Scaffold V4 software.

### Purification of His_6_-tagged EccD_5_1–131.

The portion of *eccD*_*5*_ encoding a predicted soluble cytoplasmic domain from residues 1 to 131 (EccD_5_1–131) was amplified from M. tuberculosis Erdman genomic DNA by PCR with primers His-eccD5_F1 and His-eccD5_R1 and cloned into plasmid pET28b+ between the NdeI and HindIII restriction enzyme sites to generate pET28-His_6_EccD_5_, encoding EccD_5_1–131 with an N-terminal His_6_ tag. The pET28-His_6_EccD_5_ plasmid was introduced into E. coli BL21(DE3), and the protein was purified by Ni^2+^-nitrilotriacetic acid (NTA) affinity chromatography (Qiagen) as previously described for PPE41-His_6_ (37, 38). Briefly, purified protein was bound to the column under native conditions in 20 mM HEPES buffer, 300 mM NaCl, pH 7.8, and eluted in 20 mM HEPES buffer, 500 mM NaCl, pH 7.8, containing 50 to 150 mM imidazole. Purified His_6_EccD_5_ was concentrated through a 5-kDa-cutoff Amicon Ultra centrifugal filtration unit (Millipore), and then contaminant proteins were removed by fast-protein liquid chromatography (FPLC) using a BioLogic DuoFlow apparatus (Bio-Rad). Protein was bound to an Enrich Sec 650 (Bio-Rad) column and eluted in phosphate-buffered saline (PBS) using an isocratic flow at a rate of 500 µl/min. Protein which eluted at 0.02 absorbance unit was collected and pooled.

### Antiserum production.

Polyclonal antisera against purified His_6_-EccD_5_1–131 were generated in rabbits by Pierce custom antibodies (Thermo Scientific) using TiterMax Gold adjuvant (Sigma). Polyclonal rabbit antisera against EsxN and PPE41 were previously described (38).

### Preparation of membrane vesicles.

Thirty-milliliter M. tuberculosis cultures were grown in Sauton’s medium as previously described for analysis of secreted proteins (22). MV were isolated from culture supernatants as previously described, with minor modifications (2). Briefly, cells were pelleted by centrifugation (4,700 × *g*, 15 min, 4°C), and supernatants were sequentially filtered through 0.45-µm and 0.22-µm Millex syringe filters (Millipore). The supernatants were centrifuged (4,000 × *g*, 15 min, 4°C) to remove remaining cellular debris and then concentrated to approximately 1 ml using 100-kDa-cutoff Amicon Ultra centrifugal filtration devices (Millipore). The filtrates were further processed to concentrate secreted proteins using a 5-kDa-cutoff Amicon Ultra centrifugal filtration device, as previously described (22). The 100-kDa filter retentates were then centrifuged at 100,000 × *g* (75 min, 4°C) to pellet MV. The MV pellets were resuspended in 500 µl PBS containing Complete EDTA-free protease inhibitors (Roche).

### Western blotting.

Cellular and secreted proteins were isolated from M. tuberculosis cultures grown in Sauton’s medium as previously described, with minor modifications (22). Briefly, bacteria were pelleted and culture supernatants were filter sterilized before MV were purified and secreted proteins were concentrated as described above. Pellets were resuspended in PBS containing Complete EDTA-free protease inhibitors (Roche) and subjected to bead beating to lyse the cells before they were filtered to remove intact cells. Cellular and secreted proteins were quantified via a bicinchoninic acid (BCA) assay (Pierce BCA protein assay kit; ThermoFisher). Five micrograms of cellular and secreted proteins was separated by SDS-PAGE and transferred to 0.2-µm nitrocellulose membranes (Bio-Rad) as previously described (22). The protein concentration in MV preparations was below the limit of detection of the BCA assay, so 20 µl of MV samples, corresponding to 1.2 ml of culture, was separated by SDS-PAGE. Membranes were blocked in PBS with 0.1% Tween 20 (PBST) containing 5% nonfat milk powder at room temperature for 1 to 2 h. Membranes were washed in PBST and then probed overnight at 4°C with primary antisera diluted in PBST containing 2.5% nonfat milk powder. Primary antisera were used at the following dilutions: mouse anti-KatG, 1:1,000; mouse anti-LpqH, 1:500; mouse anti-PstS1, 1:1,000; mouse anti-GroEL2, 1:1,000; rabbit anti-ModD, 1:25,000; rabbit anti-PPE41, 1:1,000; rabbit anti-EsxN 1:1,000; rabbit anti-EccD_5_, 1:1,000; and rabbit anti-VirR, 1:10,000. Membranes were again washed in PBST before incubation for 1 to 2 h at room temperature with the appropriate secondary antibody (either goat anti-rabbit or rabbit anti-mouse antibody conjugated to horseradish peroxidase; Sigma) diluted 1:30,000 (1:5,000 when used to detect VirR) in PBST containing 2.5% nonfat milk powder. Membranes were washed again in PBST, and bands were detected using SuperSignal West Pico chemiluminescent substrate (Thermo Scientific). Blots were imaged using an Odyssey Fc imaging system (LI-COR), and protein abundance was analyzed using Image Studio software (LI-COR).

### Vesicle quantification.

Filter-sterilized culture supernatants from M. tuberculosis strains grown in Sauton’s medium without Tween 80 were analyzed with a NanoSight instrument to determine vesicle numbers and sizes. Culture filtrates were diluted in a 1-ml final volume of 1× PBS (Corning) to a concentration acceptable for analysis by the NanoSight NS300 (Malvern Instruments, United Kingdom). Triplicate videos of each sample were taken at 24.5°C in light scatter mode using the equipped 532-nm green laser and a syringe pump. Particle displacement was detected with a camera level of 14, and analyses were performed using NanoSight 3.0 or 3.1 software and a threshold of between 3 and 5. Triplicate video statistics were averaged for each sample. A control culture of each strain grown in Sauton’s medium with Tween 80 was serially diluted and plated on 7H10 medium in duplicate. Colonies were counted after 3 weeks of incubation at 37°C to determine numbers of CFU per milliliter, and the number of particles per milliliter was normalized to this value to determine the number of vesicles per CFU.

### EM of purified vesicles.

All reagents were electron microscopy (EM) grade and were purchased from Electron Microscopy Supply Co. Transmission electron microscopy (TEM) was used to visualize MV purified from 600-ml cultures grown as described above. Formvar-coated copper grids were floated on 20-µl drops of purified MV solutions for 20 min. The grids were washed in water and then floated on 1% uranyl acetate for 30 s. The grids were washed again before being imaged on a FEI Tecnai Spirit Bio-Twin.

### Construction of *virR* deletion and overexpression strains.

Sequences of all primers used for cloning and strain construction are provided in Table S2 in the supplemental material. A vector for the deletion of base pairs 252 to 306 of *virR* was constructed in the allelic-exchange plasmid pJG1100, which contains the *aph* (kanamycin [Kan] resistance), *hyg* (hygromycin [Hyg] resistance), and *sacB* (sucrose sensitivity) markers (36). Regions of the M. tuberculosis genome ~900 bp 5′ and 3′ of the deletion site were amplified by PCR with primers virR_F2/virR_R6 and virR_F6/virR_R3. The virR_R6 primer to amplify the 5′ region of *virR* was designed with an AvrII restriction site in frame with the *virR* start codon, and the virR_F6 primer used to amplify the 3′ region of *virR* was designed with an AvrII restriction site in frame with the stop codon. The resulting construct encodes a copy of *virR* lacking 54 bp. The PCR products were cloned into PCR2.1-TOPO (Invitrogen) and sequenced. The 5′ and 3′ homology regions were subsequently removed from the pCR2.1 vector by digestion with PacI/AvrII or AvrII/AscI, respectively, and cloned in pJG1100 digested with PacI/AscI. The resulting pJG-Δ*virR* vector was confirmed by sequencing. Plasmids for the overexpression of FLAG-tagged *virR* (p*virR*) were previously described (8) and generously provided by Carl Nathan.

10.1128/mBio.00778-18.6 TABLE S2  Oligonucleotide primers used for cloning, strain construction, or qRT-PCR. Download TABLE S2, DOCX file, 0.1 MB. Copyright © 2018 White et al. 2018 White et al. This content is distributed under the terms of the Creative Commons Attribution 4.0 International license.

The pJG-Δ*virR* and p*virR* vectors were introduced into WT and Δ*pstA1* bacteria by electroporation as previously described (23). Transformants containing p*virR* were selected by plating them on complete 7H10 containing 50 µg/ml Hyg. The presence of p*virR* was confirmed by PCR with the primer pair pDE43_F/pDE43_R. Transformants containing pJG-Δ*virR* were selected by plating them on complete 7H10 containing 50 µg/ml Hyg and 15 µg/ml Kan. Integration of pJG-Δ*virR* at the *virR* locus was confirmed using primers virR_F4/PJGR and PJGF/virR_R4. Transformants that had integrated the plasmid were grown in complete 7H9 medium before serial dilution and plating on complete 7H10 medium containing 2% sucrose to counterselect the pJG-Δ*virR* plasmid. Sucrose-resistant isolates were screened by PCR with the primer pair virR_F4/virR_R4 to ensure loss of the pJG-Δ*virR* plasmid, and the 54-bp deletion was confirmed by sequencing.

### Cloning of *eccD*_*5*_ deletion and conditional expression vectors.

A vector for deletion of *eccD*_*5*_ was constructed in the allelic-exchange plasmid pJG1100. Regions of the M. tuberculosis genome ~800 bp 5′ and 3′ of *eccD*_*5*_ were amplified by PCR with primer pairs eccD5F1/eccD5R1 and eccD5F2/eccD5R2. The eccD5R1 primer to amplify the 5′ region was designed with an AvrII restriction site in frame with the *eccD*_*5*_ translation start codon. The eccD5F2 primer to amplify the 3′ region was designed with an AvrII restriction site in frame with the *eccD*_*5*_ stop codon. To avoid polarity of the final construct upon expression of *mycP*_*5*_, which overlaps the *eccD*_*5*_ gene at the 3′ end, the eccD5R2 primer was designed 5′ of the predicted *mycP*_*5*_ start codon. The resulting construct encodes the first 5 amino acids of EccD_5_ fused in frame to the last 16 amino acids of EccD_5_. The PCR products were cloned in pCR2.1-TOPO (Invitrogen) and sequenced. The 5′ and 3′ homology regions were subsequently removed from the pCR2.1 vector by digestion with PacI/AvrII or AvrII/AscI, respectively, and cloned in pJG1100 digested with PacI/AscI. The resulting pJG-Δ*eccD*_*5*_ vector was confirmed by sequencing.

For tetracycline-inducible expression, *eccD*_*5*_ was cloned in pTIC10a, a derivative of the integrative pTIC6 vector (39), which contains a codon-optimized Tet repressor expressed under the control of the constitutive mycobacterial *groEL* promoter, the *aph* Kan resistance marker, and the P_*smyc*_-*tetO* mycobacterial promoter and Tet operator 5′ of the multicloning site. Full-length *eccD*_*5*_ was amplified from M. tuberculosis Erdman genomic DNA by PCR with primers Tet-eccD5F and Tet-eccD5R, cloned in pCR2.1-TOPO (Invitrogen), and sequenced. The *eccD*_*5*_ insert was removed by digestion with HindIII and EcoRI and cloned in similarly digested pTIC10a. The resulting pTIC-*eccD*_*5*_ vector was confirmed by sequencing.

A vector for tetracycline-repressible expression of *eccD*_*5*_ was constructed as previously described using Gateway vectors generously provided by Dirk Schnappinger (40). *eccD*_*5*_ was amplified using primers eccD5_P1, which adds an *attB2* site upstream of the *eccD*_*5*_ promoter sequence, and eccD5_P4, which adds an *attB3* site downstream of the *eccD*_*5*_ stop codon, before being cloned into the pDO23A entry vector via a BP Gateway reaction (Gateway BP clonase II enzyme mix; ThermoFisher) to create pEN23A-*eccD*_*5*_. The final pGMCH-T38S38-750-*eccD*_*5*_ vector was constructed via an LR Gateway reaction (Gateway LR clonase II enzyme mix; ThermoFisher) using plasmids pDE43-MCH, pEN41A-T38S38, pEN12A-P750, and pEN23A*-eccD*_*5*_ and confirmed by sequencing. A similar strategy was used to construct a vector for the expression of *eccD*_*5*_-DAS containing the DAS+4 degradation tag (29). *eccD*_*5*_-DAS was amplified using primers eccD5_P1, eccD5_P2, and eccD5_P3. The eccD5_P2 and eccD5_P3 primers were used in sequential PCRs to add the DAS+4 tag with a stop codon and introduce an *attB3* site as described previously (40). Gateway reactions to clone *eccD*_*5*_-DAS into pDO23A and to construct the final pGMCH-T38S38-750-*eccD*_*5*_-DAS plasmid were performed as described above, and the final plasmid was confirmed by sequencing.

### Construction of a conditional *eccD*_*5*_ depletion strain.

The pTIC-*eccD*_*5*_ vector was introduced into WT and Δ*pstA1* mutant M. tuberculosis by electroporation as described previously (23), and transformants were selected by plating them on complete 7H10 agar containing 15 µg/ml Kan. The presence of the plasmid in transformants was verified by PCR with the primer pair pTseqF/Q95R1. The resulting WT pTIC-*eccD*_*5*_ and Δ*pstA1* pTIC-*eccD*_*5*_ strains were subsequently electroporated with pJGΔ*eccD*_*5*_, and transformants were selected on 7H10 agar containing 50 µg/ml Hyg and 15 µg/ml Kan. Integration of the pJG-Δ*eccD*_*5*_ vector at the *eccD*_*5*_ locus in transformants was confirmed by PCR using primers Q94F1/mycP5R1 and Rv1794_3′F/Q96R1 to detect integration via the 5′ and 3′ regions of homology, respectively. Isolates in which the plasmid integrated site specifically were then grown in 7H9 supplemented with 15 µg/ml Kan and 50 ng/ml anhydrotetracycline hydrochloride (ATc; Sigma) to induce *eccD*_*5*_ expression from pTIC-*eccD*_*5*_ and allow excision of pJGΔ*eccD*_*5*_. The culture was serially diluted and plated on 7H10 agar containing 15 µg/ml Kan, 100 ng/ml ATc, and 2% sucrose to counterselect the pJGΔ*eccD*_*5*_ plasmid. Sucrose-resistant isolates were patched to 7H10 medium containing 50 µg/ml Hyg to identify those that had excised pJGΔ*eccD*_*5*_. Hyg-sensitive colonies were grown in 7H9 medium containing 15 µg/ml Kan and 50 ng/ml ATc and tested by PCR for the Δ*eccD*_*5*_ mutation using primers Q94F1 and Q96R1. These strains were designated *eccD*_*5*_ Tet-ON and Δ*pstA1 eccD*_*5*_ Tet-ON.

The pGMCH-T38S38-750-*eccD*_*5*_ vector was introduced into *eccD*_*5*_ Tet-ON and Δ*pstA1 ΔeccD*_*5*_ Tet-ON bacteria by electroporation, and transformants were selected on complete 7H10 medium containing 50 µg/ml Hyg. Transformants were patched onto 7H10 medium containing 50 µg/ml Hyg and 7H10 medium containing 15 µg/ml Kan. Colonies that grew in the presence of Hyg but not in the presence of Kan were screened by PCR to ensure that the strains harbored the *hyg* cassette (primers hygro_forward and hygro_reverse) and the pGMCH-*eccD*_*5*_ vector (primers pGMCH_D5_F and pGMCH_Rev) and had lost the pTIC10a-*eccD*_*5*_ vector via a plasmid swap (primers pTIC_KanR_For and pTIC_KanR_Rev). These strains were designated *eccD*_*5*_ Tet-OFF and Δ*pstA1 eccD*_*5*_ Tet-OFF.

### Growth curves.

Frozen bacterial stocks were inoculated into complete 7H9 medium and grown to mid-logarithmic phase (optical density at 600 nm [OD_600_] = 0.6). Cultures were diluted to an OD_600_ of 0.05 in 7H9 medium with and without 100 ng/ml ATc, and OD_600_ measurements were taken daily for 15 days. Every 3 days, aliquots of each culture were serially diluted, plated on 7H10 agar, and incubated at 37°C for 3 to 4 weeks to determine numbers of viable CFU.

### qRT-PCR.

Bacteria were inoculated into 7H9 medium at an OD_600_ of 0.1, and ATc (100 ng/ml) was added where indicated in the figures. Cultures were grown at 37°C with aeration to late-logarithmic phase (OD_600_s = 0.6 to 0.8) before extraction of RNA and reverse transcription to cDNA as previously described (22, 23). Quantitative PCR primers to amplify an internal region of genes of interest (*eccD*_*5*_, *espG*_*5*_, *lpqH*, *virR*, *sigA*) were designed with similar melting temperatures (58 to 60°C) using Primer Express software (Applied Biosystems) or ProbeFinder Assay Design software (Roche). Sequences of these primers can be found in Table S2. qRT-PCRs were carried out on a LightCycler 480 (Roche), and gene copy numbers were normalized to that of *sigA* as previously described (22).

### Statistical analysis.

All statistical analyses were performed using GraphPad Prism6 software. *P* values of <0.05 were considered significant. Statistics for Fig. 2B, 3B, 4B, and 6B were calculated by ordinary one-way analysis of variance (ANOVA) with Holm-Sidak’s multiple-comparison test applied *post hoc*. Statistics for Fig. 3C, 5A, and 5B were calculated by unpaired *t* test between the indicated strains.
