# Supplementary material for: Mycobacterium tuberculosis Pst/SenX3-RegX3 Regulates Membrane Vesicle Production Independently of ESX-5 Activity
Source: mBio. 2018 Jun 12;9(3):e00778-18. doi: 10.1128/mBio.00778-18 (PMC6016242; doi:10.1128/mBio.00778-18)
Supplement: FIG S1 [file mbo003183934sf1.pdf]

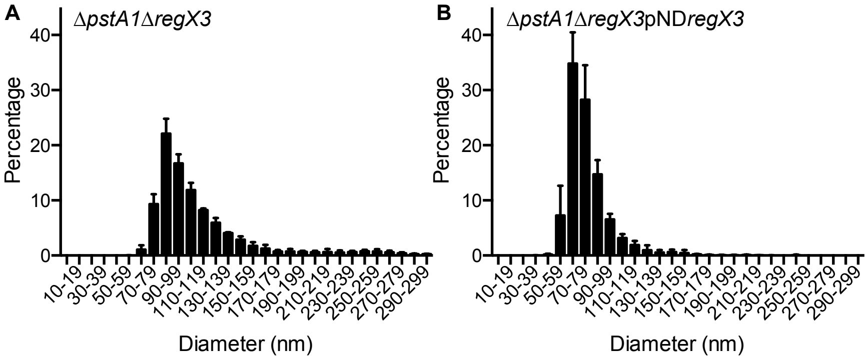

Figure S1: The Δ*pstA1*Δ*regX3* and Δ*pstA1*Δ*regX3*pND*regX3* strains produce membrane vesicles. Δ*pstA1*Δ*regX3* and Δ*pstA1*Δ*regX3*pND*regX3* strains were grown for 5 days in Sauton's complete medium without Tween-80. Nanoparticle tracking analyses were performed on culture supernatants and the resulting particle sizes were binned in 10 nm increments.
