# Supplementary material for: Mycobacterium tuberculosis Pst/SenX3-RegX3 Regulates Membrane Vesicle Production Independently of ESX-5 Activity
Source: mBio. 2018 Jun 12;9(3):e00778-18. doi: 10.1128/mBio.00778-18 (PMC6016242; doi:10.1128/mBio.00778-18)
Supplement: FIG S2 [file mbo003183934sf2.pdf]

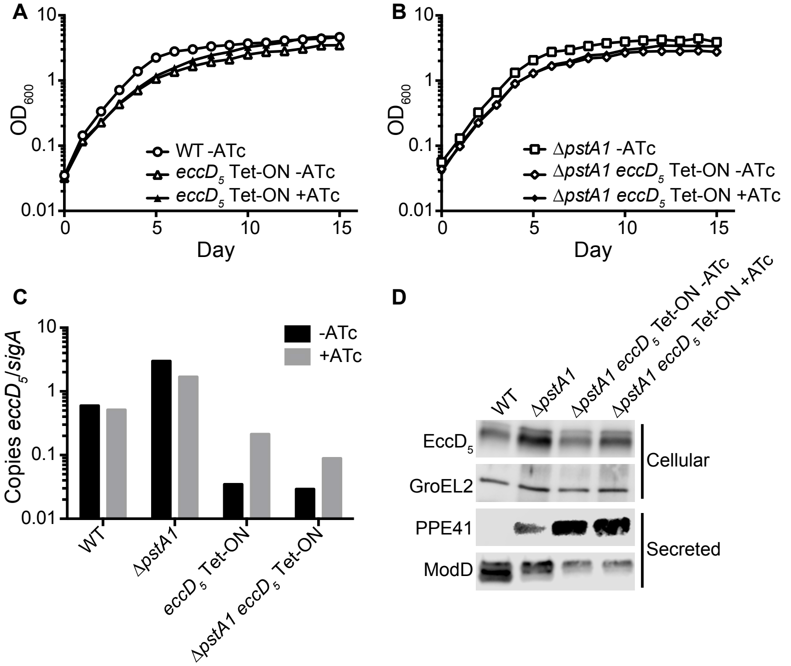

Figure S2: Residual *eccD*$_5$ expression in uninduced *eccD*$_5$ Tet-ON strains is sufficient for protein secretion through ESX-5. Kanamycin (15 µg/ml) was added to all *eccD*$_5$ Tet-ON cultures to maintain plasmid integration. (A&B) Wild-type *M. tuberculosis* Erdman (WT), Δ*pstA1*, *eccD*$_5$ Tet-ON, and Δ*pstA1 eccD*$_5$ Tet-ON strains were inoculated in 7H9 complete medium at an OD_{600} of 0.05 and grown at 37˚C with aeration. Anhydrotetracycline hydrochloride (ATc; 50 ng/ml) was added every 3 days to indicated cultures. Growth was monitored by daily OD_{600} measurements. (C) Transcript abundance of *eccD*$_5$ relative to *sigA* was determined by quantitative RT-PCR for the WT, Δ*pstA1*, *eccD*$_5$ Tet-ON, and Δ*pstA1 eccD*$_5$ Tet-ON strains grown to mid-logarithmic phase in 7H9 complete medium with and without ATc. (D) The WT, Δ*pstA1*, *eccD*$_5$ Tet-ON, and Δ*pstA1 eccD*$_5$ Tet-ON strains were grown for 5 days in Sauton's complete medium without Tween-80. ATc (50 ng/ml) was added to the indicated cultures to induce *eccD*$_5$. Equivalent amounts of cellular (5 µg) and secreted (5 µg) proteins were analyzed by Western blot to detect the indicated proteins.
