# Supplementary material for: Mycobacterium tuberculosis Pst/SenX3-RegX3 Regulates Membrane Vesicle Production Independently of ESX-5 Activity
Source: mBio. 2018 Jun 12;9(3):e00778-18. doi: 10.1128/mBio.00778-18 (PMC6016242; doi:10.1128/mBio.00778-18)
Supplement: FIG S3 [file mbo003183934sf3.pdf]

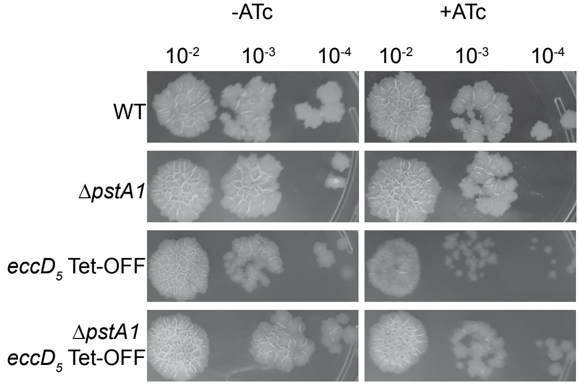

Figure S3: Repression of *$eccD_5$* results in small colony morphology. Wild-type *M. tuberculosis* Erdman (WT), Δ*pstA1*, *$eccD_5$* Tet-OFF, and Δ*pstA1* *$eccD_5$* Tet-OFF strains were inoculated in 7H9 complete medium at an OD_{600} of 0.05. Cultures were serially diluted and 10 µl of the indicated dilutions were spot plated on 7H10 complete medium ± ATc (100 ng/ml).
