# Supplementary material for: Mycobacterium tuberculosis Pst/SenX3-RegX3 Regulates Membrane Vesicle Production Independently of ESX-5 Activity
Source: mBio. 2018 Jun 12;9(3):e00778-18. doi: 10.1128/mBio.00778-18 (PMC6016242; doi:10.1128/mBio.00778-18)
Supplement: FIG S4 [file mbo003183934sf4.pdf]

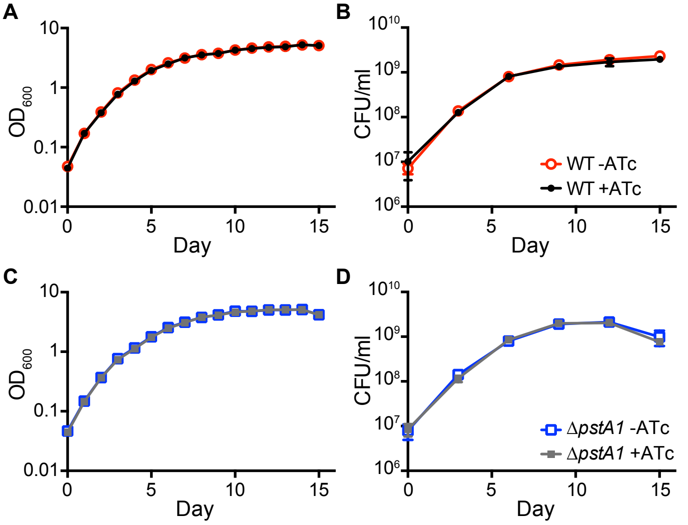


**Figure S4: ATc does not affect the growth of WT or Δ*pstA1* bacteria.** Wild-type *M. tuberculosis* Erdman (WT) and Δ*pstA1* strains were inoculated in 7H9 complete medium at an $OD_{600}$ of 0.05 and grown at 37˚C with aeration. Anhydrotetracycline hydrochloride (ATc; 100 ng/ml) was added at Day 0 and Day 7 as indicated. Growth was monitored by daily $OD_{600}$ measurements (A&C) and by plating serially diluted cultures on 7H10 medium to determine viable CFU/ml on Days 0, 3, 6, 9, 12, and 15 (B&D).
