# Supplementary material for: Mycobacterium tuberculosis Pst/SenX3-RegX3 Regulates Membrane Vesicle Production Independently of ESX-5 Activity
Source: mBio. 2018 Jun 12;9(3):e00778-18. doi: 10.1128/mBio.00778-18 (PMC6016242; doi:10.1128/mBio.00778-18)
Supplement: TABLE S2 [file mbo003183934st2.docx]

**Table S2: Oligonucleotide primers used for cloning, strain construction or qRT-PCR.**

| Name | Purpose | Sequence 5’-3’^a^ |
| --- | --- | --- |
| His-eccD5_F1 | Cloning His_6_-EccD_5_1-131 | TTCATATG**ATG**ACCGCAGTAGCTGACG |
| His-eccD5_R1 | Cloning His_6_-EccD_5_1-131 | ATAAGCTT**TTA**GAACCGCTTGCTGAGATCC |
| eccD5F1 | 5’ ∆*eccD_5_* | GATTAATTAAACGGGTTGGCAATGACATCAC |
| eccD5R1 | 5’ ∆*eccD_5_* | GTCCTAGGAGCTACTGCGGT**CAT**CGTCCT |
| eccD5F2 | 3’ ∆*eccD_5_* | GTCCTAGGCCGCTGGCGTTGTGGTTGAT |
| eccD5R2 | 3’ ∆*eccD_5_* | TCGGCGCGCCGGCGGATGGAGATTATCTCGAC |
| Tet-eccD5F | Cloning *eccD_5_* Tet-ON | CAAAGCTTTCGGTGCCTACACCGACG |
| Tet-eccD5R | Cloning *eccD_5_* Tet-ON | CAGAATTCGGCAATGGTCGCGGTACC |
| pTseqF | Check Tet-ON strains | CATCCCGGCGTTGATCTGTG |
| Q95R1 | Check Tet-ON strains/*eccD_5_* qRT-PCR | AGCCTCACCGAGCTCTCTGA |
| Q94F1 | Check ∆*eccD_5_*/*espG_5_* qRT-PCR | CGTTGCGCCTGAGTTACGTT |
| mycP5R1 | Check ∆*eccD_5_* | GTCCTAGGACACCACGACCTGCTGCTAC |
| Rv1794_3’F | Check ∆*eccD_5_* | TTCCTAGGACACACAGCAGAGTATGACGC |
| Q96R1 | Check ∆*eccD_5_* | CCATGTTGGCAGCATGCA |
| eccD5_P1 | Gateway BP reaction | GGGG*ACAGCTTTCTTGTACAAAGTGG*AGGGGGTACAGGACG**ATG**AC |
| eccD5_P2 | Gateway BP reaction | CGCGTAGTTCTCGGAGTAGTTCTCGTCGTTGGCGGCCCGGTACCGAATCGCTGCATAG |
| eccD5_P3 | Gateway BP reaction | GGGG*ACAACTTTGTATAATAAAGTTG***CTA**GCTGGCGTCCGCGTAGTTCTCGGAGTAGTTC |
| eccD5_P4 | Gateway BP reaction | GGGG*ACAACTTTGTATAATAAAGTTG***CTA**CCGGTACCGAATCGCTG |
| pTIC_KanR_ For | Check Tet-OFF strains | CAACGGGAAACGTCTTGCTCGA |
| pTIC_KanR_ Rev | Check Tet-OFF strains | GCGATTCCGACTCGTCCAACATC |
| Hygro_for | Check Tet-OFF strains | AGCCCGACCCGGTCATCAAG |
| Hygro_rev | Check Tet-OFF strains | GGGAGTCTCCCGCATAGACG |
| pGMCH_D5_F | Check Tet-OFF strains | CATCGGTGCGTGATGTGCTG |
| pGMCH_Rev | Check Tet-OFF strains | GCCAGCTAGGCCATCGAATTCC |
| Q95F1 | *eccD_5_* qRT-PCR | CCAACGCCCCAGTTTCG |
| Q94R1 | *espG_5_* qRT-PCR | CTCGTTTAGCCAGTCATTGGAA |
| lpqH_F1 | *lpqH* qRT-PCR | TCGACGGTAAGGACCAGAAC |
| lpqH_R1 | *lpqH* qRT-PCR | CCCAACGGACTTCACCTC |
| virR_F1 | *virR* qRT-PCR | GATGGTGCTGTTGTTTCTCG |
| virR_R1 | *virR* qRT-PCR | GTTCGGAGACGAACCCAGT |
| virR_F2 | Cloning ∆*virR* | ATTTAATTAAGGTCGCAGTGTGTAAGAC |
| virR_R3 | Cloning ∆*virR* | ATGGCGCGCCAACCAATGACACCGCACGC |
| virR_F4 | Check ∆*virR* strains | GCGATCAACTGGGCGAGATC |
| virR_R4 | Check ∆*virR* strains | GAGCCTGCGAAATCAATTCGGG |
| virR_F6 | Cloning ∆*virR* | ATCCTAGGTTCACGGTCACCGACGTTGG |
| virR_R6 | Cloning ∆*virR* | ATCCTAGGGATGTTGTAGACGCGCACCTCG |
| sigA_F5 | *sigA* qRT-PCR | CTCAAACAGATCGGCAAGGT |
| sigA_R5 | *sigA* qRT-PCR | CGCTAAGCTCGGTCATCAG |
| pDE43_F | Check p*virR* strains | CATGTGAGCAAAAGGCCAGC |
| pDE43_R | Check p*virR* strains | GCGTCAGACCCCGTAGAAAAG |
| PJGF | Check ∆*virR* strains | GTGGACCTCGACGACCTC |
| PJGR | Check ∆*virR* strains | AAATGCCGATATCCTATTGGC |

^a.^ Restriction enzyme sites are underlined. Att sites are in italics. Start and stop codons are indicated in bold.
